# Coronary artery surgery: cardiotomy suction or cell salvage?

**DOI:** 10.1186/1749-8090-2-46

**Published:** 2007-10-25

**Authors:** Kelvin Lau, Hetul Shah, Andrea Kelleher, Neil Moat

**Affiliations:** 1 Department of Cardiac Surgery, Royal Brompton Hospital, and NHLI at Imperial College, London SW3 6NP, UK; 2 Department of Anaesthesia, Royal Brompton Hospital, and NHLI at Imperial College, London SW3 6NP, UK

## Abstract

Coronary artery bypass grafting (CABG) today results in what may be regarded as acceptable levels of blood loss with many institutions avoiding allogeneic red cell transfusion in over 60% of their patients. The majority of cardiac surgeons employ cardiotomy suction to preserve autologous blood during on-pump coronary artery bypass surgery; however the use of cardiotomy suction is associated with a more pronounced systemic inflammatory response and a resulting coagulopathy as well as exacerbating the microembolic load. This leads to a tendency to increased blood loss, transfusion requirement and organ dysfunction. Conversely, the avoidance of cardiotomy suction in coronary artery bypass surgery is not associated with an increased transfusion requirement. There is therefore no indication for the routine use of cardiotomy suction in on-pump coronary artery surgery.

## Introduction

Coronary artery bypass grafting (CABG) today results in what may be regarded as acceptable levels of blood loss with many institutions avoiding allogeneic red cell transfusion in over 60% of their patients [1,2]. The results of on-pump coronary artery bypass surgery are excellent in terms of early mortality [3]. However there remains significant associated morbidity, including bleeding and secondary organ dysfunction such as neurological and renal impairment.

The cardiotomy suction apparatus was introduced in the 1960s as an extension of the intracardiac vent to allow blood shed into the operative field to be returned to the cardiopulmonary bypass (CPB) circuit. The aim was to reduce blood loss and hence the need for allogeneic blood transfusions with its known risk of mortality, other sequelae [4-6] and cost. Recent evidence however, suggests that the return of shed blood by cardiotomy suction does not reduce blood loss or blood transfusion requirement in CABG [7,8]. On the other hand, it has been shown to increase the burden of microembolisation and potentiates the systemic inflammatory response.

## Pericardial shed blood

Blood which has extravasated into the pericardial or pleural cavities and which is subsequently aspirated by cardiotomy suction differs markedly from intravascular blood or blood within a closed CPB circuit. Surgical trauma from opening the chest results in substantial tissue damage and release of tissue factor [9,10]. Exposure of blood to tissue factor results in rapid activation of the extrinsic pathway of the coagulation system with release of thrombin and fibrin. In addition, tissue plasminogen activator release stimulates fibrinolysis. Analysis of pericardial shed blood shows high concentrations of markers of clotting activation and fibrinolysis [11,12] and a low heparin concentration [7]. Activation of the coagulation cascade inevitably results in activation of the other inflammatory cascades. High levels of inflammatory markers such as TNF-alpha, IL-6, IL-8 and have been identified in pericardial shed blood [8,13,14].

In addition to activation of the coagulation cascade, complement system and fibrinolytic pathways, platelets are activated when extravasation occurs into the pericardial cavity. This results in aggregation, degranulation and consumption of platelets, as well as the release of further vasoactive substances [9,12].

The pericardial space also contains a mixture of debris resulting from surgical trauma, most noticeably sternal marrow fat and air microbubbles [15]. Thus, shed 'blood' is far from pure and contains a substantial fraction of potential embolic substances as well as activated platelets and vaso-active mediators. It has been shown that these additional constituents are likely to adversely affect flow characteristics in the micro-circulation with shed blood having grossly abnormal flow characteristics when passed through a 5 micron filter [16].

## Cardiotomy suction

Flow within the cardiotomy suction tubing differs significantly from that in the CPB circuit. The concurrent suction of air results in highly turbulent flow with high shear stresses at the air-fluid interface. This causes cellular damage as well as being a potent activator of all the humoral cascades involved in the systemic inflammatory response. Cardiotomy suction blood therefore contains an elevated level of free haemoglobin [7,13,17] due to mechanical haemolysis. High concentrations of free haemoglobin cause platelet dysfunction and direct injury to the renal tubular cells [18]. Similarly, platelet numbers are reduced in cardiotomy suction blood through the rheological trauma [19].

## Re-transfusion of shed blood retrieved by cardiotomy suction

Retransfusing the vasoactive and activated inflammatory mediators in shed blood into the patient's circulation magnifies the systemic inflammatory response. In one study where shed blood was retransfused, the plasma levels of complement C3a, TNF-alpha, IL-6 were significantly elevated compared to when there was no retransfusion [8]. The clinical effect of this was demonstrated by a significant reduction in systemic vascular resistance (SVR) at the point of retransfusion [20]. This fall in SVR correlated to the concentration of TNF-alpha returned to the circulation.

Fat embolisation was known to be associated with cardiotomy suction as long ago as 1963 [21] when fat globules were noted in the urine of patients where the blood overflowed into the pericardium at operation. The authors associated this with the 'post-perfusion syndrome' and advocated discarding this shed blood. Over three decades later, Moody and colleagues demonstrated small capillary and arteriolar dilatations (SCADs) in the brains and other organs of patients who died following cardiopulmonary bypass [22], and confirmed that they were in fact fat emboli lodged within the vessels [23]. Cardiotomy suction blood is known to be saturated with fat released from the subcutaneous tissue and sternal marrow on sternotomy [24,25].

## Alternative strategies

There have been many attempts to reduce the problems associated with cardiotomy suction by using filters. These have been shown to reduce, but not eliminate the microembolic load [26,27]. Greater reductions in embolic load have been achieved with the use of serial filters [25] however filters do not have any beneficial impact upon the other problems associated with cardiotomy suction, namely derangement of coagulation and activation of inflammatory cascades.

An alternative strategy which has been employed with some success is to use controlled suction which reduces the air-fluid interface and shear stress and thereby attenuates haemolysis and the inflammatory response [19,28].

In an animal model, discarding the shed blood reduced the lipid micro-embolic load more than ten fold compared to when cardiotomy suction was used and the shed blood reinfused [23]. A number of other studies comparing the effects of cardiotomy suction plus retransfusion versus discarding the cardiotomy suction blood in primary CABG have shown a significant reduction in the systemic inflammatory response and haemolysis in the latter group [8,14,29]. Moreover, this was not associated with an increase in the transfusion requirement in the second group but rather a trend towards increased mediastinal bleeding [7,8,30], higher levels of circulating IL6, TNF and C3a [8] and increased thrombin generation, PMN elastase and beta thromboglobulin levels [14] when cardiotomy suction was used, and the shed blood reinfused.

## Intra-operative cell salvage

Red cell salvage has been in use in this country in cardiac surgery in a few institutions since 1976. Its use however became widespread following two Health Service Circulars issued by the Chief Medical Officer recommending cell salvage as a key component of the appropriate use of blood [31,32]. Most cardiac units now employ intraoperative cell salvage for complex cases and some use it routinely in all cardiac procedures requiring cardiopulmonary bypass.

Intraoperative autologous red cell salvage during cardiopulmonary bypass is an attractive alternative to cardiotomy suction. It allows the conservation of red blood cells whilst reducing the retransfusion of fat micro-emboli, activated coagulation and inflammatory markers. When the blood is aspirated from the pericardium, heparin is delivered at an appropriate rate to the tip of the suction cannula to minimize activation of coagulation. The salvaged blood is then stored in a reservoir containing additional heparinised saline prior to processing. During processing the red cells are retained in the bowl whilst the plasma, platelets, heparin, free haemoglobin, and inflammatory mediators are discarded with the wash solution. This process may be discontinuous or continuous, and the resulting red cells are finally resuspended at a haematocrit of 50 – 70% in normal saline, and reinfused.

Fat microembolic load is decreased by the cell saver by as much as 85% [25,26,33-35]. In an animal model of CPB; the processing of shed blood by a cell saver resulted in a significant reduction in the formation of SCADs [25]. Indeed there is some evidence that the continuous autotransfusion devices may now be capable of removing 100% of fat from cell salvage blood [33].

The process of cell salvage results in the activation of white blood cells leading to the release of inflammatory mediators (IL-6, C5a, C3a, terminal complement complexes). However unlike cardiotomy suction blood, the centrifugation and washing processes reduce the concentration of white blood cells by 30 – 80% and inflammatory mediators by 90 – 95% as they are discarded in the wash solution [36].

Cell salvage is not however entirely without problems; the issue of air-fluid interfaces remains, although the avoidance of "skimming" and the presence of heparin at the tip of the suction apparatus reduces the activation of the clotting and inflammatory cascades. It is also easy to understand that if very large volumes of blood are processed through a cell saver it will deplete that volume of blood of platelets and clotting factors, careful monitoring and replacement of these may be necessary.

A small number of studies have shown no adverse effect on mediastinal drainage or transfusion requirements when a cell saver is used to replace cardiotomy suction in coronary surgery [17,30]. A small randomised clinical trial (20 vs 20), demonstrated that reinfusion of cell saved blood did not increase mediastinal blood loss or blood product usage [34]. These published studies were supported by the results of an audit in our own institution (of first time CABG; 2004–05) in which there was a trend for blood loss or product usage to be higher in those patients managed with cardiotomy suction rather than cell salvage.

## Contemporary clinical studies

It is conceivable that many of the reported benefits attributed to mini-extracorporeal circulation (MECC) systems and off-pump CABG (decreased bleeding, decreased inflammatory response and reduced micro-emboli) may at least in part be attributable to the avoidance of cardiotomy suction. Recent developments in CPB designed to minimise its adverse effects resulted in MECC systems comprising of a short, reservoir-free, heparinised circuit with a centrifugal pump and membrane oxygenator [37]. The closed-circuit design without cardiotomy suction and vents eliminates any air-fluid interface, and pericardial shed blood is not reinfused. Several studies comparing MECC circuits to crude cardiopulmonary bypass circuits have found an attenuated systemic inflammatory response [38], reduced microembolisation [39] and reduced the need for red cell transfusion [40] associated with the former. It is likely that the elimination of cardiotomy suction contributed significantly to the improved outcome of these patients, and further studies are required to compare MECC directly to CPB without cardiotomy suction.

The elimination of CPB and cardiotomy suction altogether in off-pump coronary artery bypass (OPCAB) is also associated with a reduced systemic inflammatory response [41,42], lower transfusion rate [43] and fewer microemboli [44]. Whilst there is some evidence that the organ protective effects of MECC approximates that of OPCAB [45], a direct comparison between OPCAB and CPB without cardiotomy suction is required to ascertain whether the elimination of cardiotomy suction is enough to explain the difference.

## Summary

We know that conventional CPB induces a systemic inflammatory response and results in microembolism to the brain and other organs. Salvage of shed blood by cardiotomy suction exacerbates the inflammatory response and increases the load of lipid emboli to the brain (and by implication, all other capillary beds). Discarding shed mediastinal blood will attenuate these adverse effects but at the cost of losing red cells mass. Cell salvage is an attractive alternative although current evidence suggests that the complications associated with reinfusing shed blood may be attenuated rather than eliminated.

We hope that we have demonstrated that there is good evidence that CABG can be safely performed without the use of cardiotomy suction, with red cell salvage, and without an increase in blood loss or blood usage. Despite this evidence, cardiotomy suction continues to be indiscriminately used during coronary artery bypass surgery, and although in the presence of rapid significant blood loss its use is justified, its continuous application in the operative field is not.

## Conclusion

There is robust, published, clinical evidence that salvage of shed blood by cardiotomy suction and its reinfusion is deleterious to patients undergoing coronary artery bypass surgery and we suggest that there is no indication for its routine use in this group. We conclude that cell salvage represents an acceptable alternative to cardiotomy suction by attenuating the deleterious effects of the reinfusion of cardiotomy suction blood whilst preserving the red cell mass.

## List of Abbreviations

CABG – coronary artery bypass graft

CPB – cardiopulmonary bypass

MECC – mini-extracorporeal circulation

OPCAB – off-pump coronary artery bypass

PMN – polymorphonuclear

SCAD – small capillary and arteriolar dilatation

SVR – systemic vascular resistance

TNF – tumour necrosis factor

## Competing interests

The author(s) declare that they have no competing interests.

## Authors' contributions

Lau, K.K.W.: Acquisition of data, Literature search and preparation of draft of manuscript

Shah, H: Acquisiton of Data

Kelleher, A: Design of audit and preparation of draft of manuscript

Moat, N: Design of audit and preparation of draft of manuscript

All authors read and approved the final manuscript
